# Not That Heart-Stopping After All: Visuo-Cardiac Synchrony Does Not Boost Self-Face Attribution

**DOI:** 10.1371/journal.pone.0160498

**Published:** 2016-08-19

**Authors:** Giuseppina Porciello, Moritz M. Daum, Cristina Menghini, Peter Brugger, Bigna Lenggenhager

**Affiliations:** 1 Department of Psychology, ‘Sapienza’, University of Rome, Rome, Italy; 2 Social and Cognitive Neuroscience Laboratory, IRCCS, Santa Lucia Foundation, Rome, Italy; 3 Department of Psychology, University of Zurich, Zurich, Switzerland; 4 Department of Neurology, University Hospital Zurich, Zurich, Switzerland; 5 Institute of Physiology and Zurich Center for Integrative Human Physiology (ZIHP), University of Zurich, Zurich, Switzerland; Ludwig-Maximilians-Universität München, GERMANY

## Abstract

Recent experimental evidence and theoretical models suggest that an integration of exteroceptive and interoceptive signals underlies several key aspects of the bodily self. While it has been shown that self-attribution of both the hand and the full-body are altered by conflicting extero-exteroceptive (e.g. visuo-tactile) and extero-interoceptive (e.g. visuo-cardiac) information, no study has thus far investigated whether self-attribution of the face might be altered by visuo-cardiac stimulation similarly to visuo-tactile stimulation. In three independent groups of participants we presented ambiguous (i.e. morphed with a stranger's face) self-faces flashing synchronously or asynchronously with the participants’ heartbeat. We then measured the subjective percentages of self-face attribution of morphed stimuli. To control for a potential effect of visuo-cardiac synchrony on familiarity, a task assessing the attribution of a familiar face was introduced. Moreover, different durations of visuo-cardiac flashing and different degrees of asynchronicity were used. Based on previous studies showing that synchronous visuo-cardiac stimulation generally increases self-attribution of the full-body and the hand, and that synchronous visuo-tactile stimulation increases self-face attribution, we predicted higher self-face attribution during the synchronous visuo-cardiac flashing of the morphed stimuli. In contrast to this hypothesis, the results showed no difference between synchronous and asynchronous stimulation on self-face attribution in any of the three studies. We thus conclude that visuo-cardiac synchrony does not boost self-attribution of the face as it does that of hand and full-body.

## Introduction

Increasing evidence suggests that multisensory integration and neurophysiological representation of the body crucially underlie our sense of self (see e.g. [1] for a recent review). Empirical research in this field has often used paradigms which induce situations of multisensory conflicts in order to alter and study the bodily self. The most famous of these paradigms is the so-called rubber hand illusion, in which the participant's hidden hand is placed in front of the participant along with a rubber hand, and both are synchronously stroked. In this condition, the seen stroking of the rubber hand dominates the felt stroking of the participant's hidden real hand, inducing an illusory feeling of ownership over the former, as measured by questionnaires at the explicit level [2]. In addition to these explicit measures, implicit indices have been described which assess the strength of the illusion, that is among others a proprioceptive drift of the real hand towards the position of the rubber hand [2], skin conductance response to threat (e.g. [3,4]), changes in body temperature [5], local alterations in histamine reactivity [6], or altered neural activity (e.g. [7,8]). Variants of this paradigm were used to induce changes in the representation of the foot [9], the full-body [10], the tongue [11] and the face [12,13]. In the latter case, a mirror effect (“enfacement illusion” [12,13]) is created by stroking applied simultaneously to the participant's face and the face of someone sitting in front of the participant. In this situation participants typically self-attribute the seen face more during synchronous than asynchronous visuo-tactile stimulation. This is usually measured by self-reported questionnaires and by asking participants to evaluate how much of the self is present in pictures or videos of the participant's face morphed by the varying presence of another's face. While these experimental manipulations suggest that an erroneous integration of *exteroceptive* bodily signals (i.e., vision and touch) might alter several aspects of the bodily self, some exciting recent studies found that erroneous integration of extero- and interoceptive bodily signals (i.e. vision and cardiac information) might also alter the representation of the hand [14] or full-body [15]. Specifically, Aspell and collaborators [15] found that when participants view a virtual body glooming synchronously with their heartbeat, they reported higher level of self-identification with the avatar and larger drift in self-location towards the virtual body (this was not found if the virtual body was simply an object). Similarly, Suzuki and collaborators [14] used a cardiac version of the classical rubber hand illusion to show that participants self-attribute a virtual hand at a higher rate when it flashes synchronously with their heartbeat as opposed to asynchronously, and that this effect is stronger for people with higher interoceptive sensitivity. These behavioral findings suggest that interoceptive and exteroceptive signals are integrated and crucially contribute to the feeling of owning a body (body ownership) and to the feeling of localizing the body in a specific part of space (self-location). An integration of exteroceptive and interoceptive signals for the bodily self are also suggested by empirical works showing that the strength of exteroceptively-induced illusions depends on Interoceptive Accuracy [16,17], and by theoretical work according to which internal states and visceral signals are crucial for self-representation [18,19].

At the neuroanatomical level, such integration might be plausibly located in the anterior insular cortex [20]. The anterior insula is a core region for interoceptive processing. Through the tight interrelation with cortical (mainly cingulate and prefrontal cortex) and subcortical (amygdala and ventral striatum) areas, interoceptive representations are integrated in cross-modal processing. They are also thought to influence higher-order perceptual and conceptual representations related to the bodily self (see [21] for a review).

Here, in three independent studies, we investigated whether visuo-cardiac synchronicity might directly influence another basic and crucial aspect of the bodily self: self-face attribution. Because visuo-cardiac synchronicity has previously been shown to increase self-attribution of a virtual hand and a virtual full-body [14,15], we expected higher self-attribution of ambiguous (i.e. morphed with a stranger's face) self-faces when presented as flashing synchronously with one’s own heartbeat, similarly to the synchronous seen touch observed during the enfacement illusion [12,13]. Furthermore, since susceptibility to visuo-tactile and visuo-cardiac illusions depends on individual Interoceptive Accuracy [14,16,17], we expected higher self-face attribution in participants with higher Interoceptive Accuracy (i.e. people achieving high scores to objective behavioural tests about heartbeat detection) and/or Interoceptive Sensibility (i.e. people scoring high to self-evaluation of subjective interoception, see [22] for the distinction in terminology), as well as in participants who localize their self in regions closer to the heart (measured through a bodily Self Localization task [23]). Moreover, we expected visuo-cardiac synchronicity to specifically increase self-face attribution rather than enhance a general sense of familiarity with ambiguous facial stimuli. To differentiate a general increase in familiarity from a bias in self-attribution, we introduced a control task in which we measured, in the same experimental conditions and setting, changes in the percentage of familiar face attribution (namely, friends in Study 1 and 2, and famous persons in Study 3).

In Study 1 and 2, morphed faces flashed for 8 seconds, synchronously or not with the participant's heartbeat. The level of asynchronicity was modified between the studies (Study 1: 90% or 110% vs. Study 2: 75% or 125% vs. Study 3: 50% or 150%). In light of null results for Study 1 and 2 (see below), we increased the visuo-cardiac stimulation in Study 3 to two minutes, making it more comparable to enfacement studies (see for example [12,13]). In line with this methodological change, two previous studies used this longer duration and reported a significant increase in self-attribution induced by visuo-cardiac synchronicity [14,15]. Moreover, recent results [24] have shown that changes of self-face representation induced by a visuo-tactile stimulation may take some seconds to appear with considerable variability across participants. Longer durations of the visuo-cardiac stimulation may thus be necessary.

## Study 1

### Materials and Methods

#### Participants

In the first study, a statistical power analysis was performed for sample size estimation (G*Power 3.1, [25]) by deciding a priori a medium (using Cohen’s criteria [26]) effect size of 0.30 (similar to the one found in [15]), an alpha of 0.05, a power of 0.95, and a unique group with 3 within-subjects measurements, namely the comparison of the three ‘Flashing Rates’ (Synchronous; Asynchronous and Heartbeat-Independent). The power calculation recommended a sample size of *N* = 31. We thus stopped the data collection after reaching a sample size of 32 females, namely 16 pairs of friends (age: 21.88 ± 2.71years; *M* ± *SD*). All participants, except two, were right-handed. Written informed consent was obtained from, and compensation was provided to, each participant. The original sample included three more participants who were excluded because they became aware of the experimental manipulation (*N* = 2), or the electrocardiographic signal was too noisy (*N* = 1). The study was approved by the local Ethics Committee of the Faculty of Arts and Social Sciences, University of Zurich.

#### Procedures

**Fig 1:**
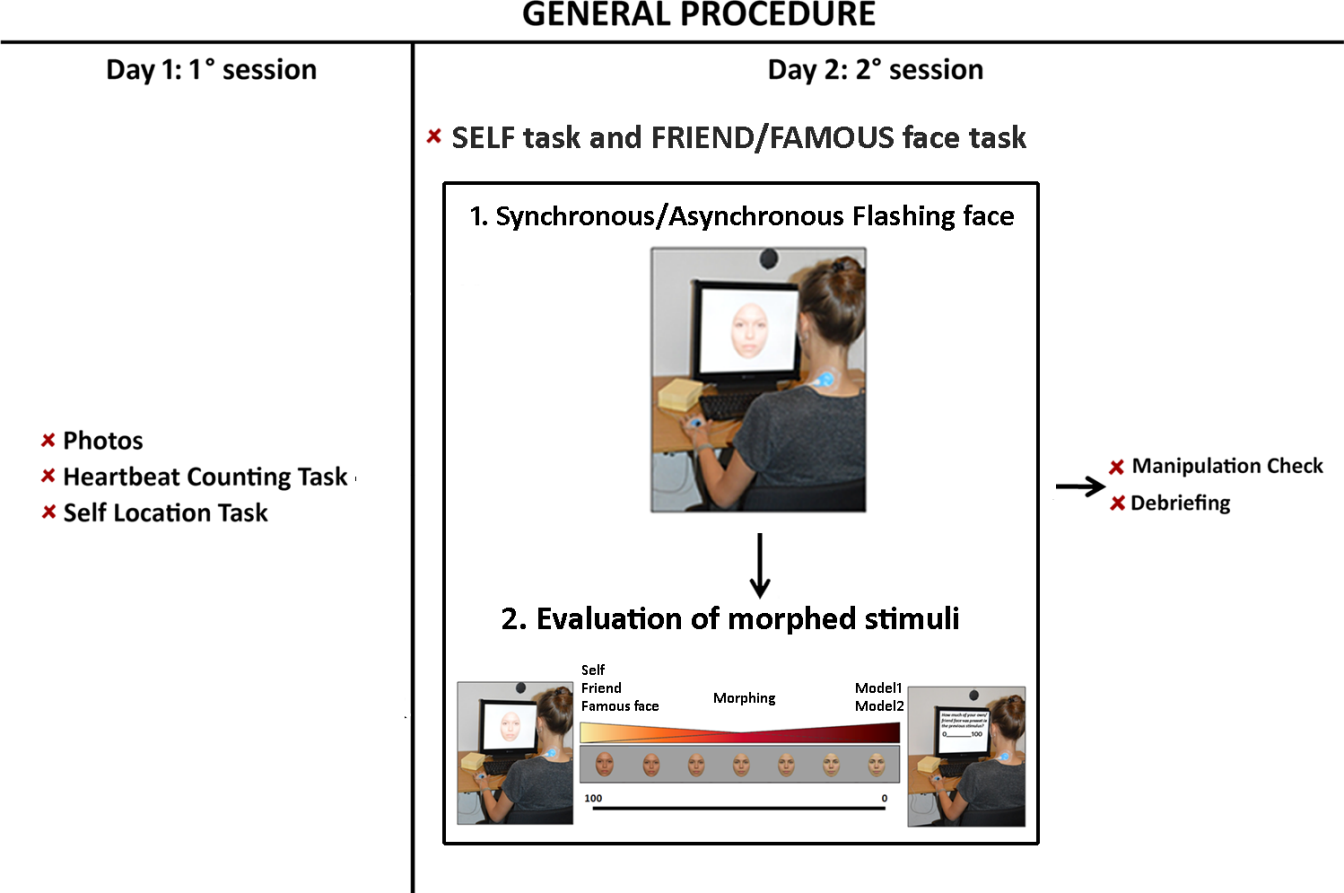
Overview of the general experimental procedure used in Study 1, 2 and 3. All studies were composed by two sessions performed in two different days, separated about one week. During the first session photos of participants’ face were taken. Moreover, participants performed: the Heartbeat Counting task; a subscale (i.e. Noticing) of the Multidimensional Assessment of Interoceptive Awareness (MAIA) and the Self Localization task. During the second session participants performed in counterbalanced order the SELF task and the FRIEND task in Study 1 and 2 and the SELF task and the FAMOUS FACE task in Study 3. During these tasks, participants observed a series of morphed faces (their own one and their friend one morphed with two unknown models) flashing on the monitor for 8 seconds (Study 1 and 2) or two single morphed faces (their own one and a famous character one morphed with two unknown models) for 2 minutes (Study 3). Immediately after the flashing faces participants were instructed to rate on visual analogue scale (VAS), how much of the own/friend or famous person’s facial features they perceived in a list of morphed stimuli. The SELF and FRIEND/FAMOUS task were composed by three blocks. Each block was associated to a different ‘Flashing Rate’ of stimuli presentation. At the end of the SELF and FRIEND/FAMOUS FACE task participants were asked whether they were aware of the research questions and hypotheses, then they were debriefed by the experimenter. Participant appearing in the picture gave written informed consent to publish this figure.

The study included two sessions separated by about seven days (Fig 1 shows a schema of the procedure). During the *first session*, a photo of each participant’s face was taken in controlled light settings, looking straight with a neutral expression. By serving as non-facial cues, ears and hair were removed and faces were placed in an oval shape (Adobe Photoshop®). Participants’ and friends’ faces were morphed (Abrasoft FantaMorph®) at different percentages with the photographs of two unknown females (rated equally attractive by an independent sample of 14 female participants (*t*(13) = 0.983; *p* = .343). The two females were used as models for the morphing but did not perform the experimental task. Thirty morphed pictures for the SELF and thirty for the FRIEND task were generated. The percentages of self face in the SELF task and friend face in the FRIEND task were as follows: 1%; 3%; 5%; 7%; 9%; 31%; 33%; 35%; 37%; 39%; 41%; 43%; 45%; 47%; 49%; 51%; 53%; 55%; 57%; 59%; 61%; 63%; 65%; 67%; 69%; 91%; 93%; 95%; 97% 99%. Our selection of the morphed stimuli was guided by previous experimental evidence for enfacement having the strongest effect on very ambiguous stimuli (45% self face-55% other face) [12,27]. We therefore tested whether cardiac information could directly alter the perception of the most sensitive visual morphs.

Interoceptive Accuracy was assessed by the Heartbeat Counting task [28] in which participants were asked to silently count their heartbeats during four time intervals (25s, 35s, 45s, 100s). Auditory cues indicated the start and end of the counting period. Heart rate was recorded using an ECG (e-Health, Cooking Hacks). A 0 (‘very weak’) to 1 (‘very high’) Interoceptive Accuracy-index was calculated from the measured and the counted number of heartbeats. Descriptive statistics are reported in Table 1.

**Table 1:** Descriptive statistics relative to: i) the Heartbeat Counting Task (M ± SD); ii) a subscale (i.e. Noticing) of the Multidimensional Assessment of Interoceptive Awareness (MAIA), (M ± SD) and iii) the Self Localization task [percentages of females who localized themselves within the brain region (‘Brainers’); within the torso close to the heart region (‘Hearters’); close to the belly region (‘Belliers’); or in the legs/arms (‘Other body parts’)] calculated for the whole sample of participants (Study 1; 2; 3).

Heartbeat Counting Task	MAIA	Self Localization Task			
	Noticing	Brainers	Hearters	Belliers	Other body parts
0.64 (0.16)	3.25 (0.83)	34.67%	45.33%	16.00%	4.00%

Interoceptive Sensibility (one of the three dimensions of interoception according the model proposed by [22], namely the dimension representing self-report measures of subjective interoception) was assessed by one sub-scale (i.e. Noticing) of the Multidimensional Assessment of Interoceptive Awareness (MAIA) questionnaire [29]. Descriptive statistics are reported in Table 1.

In a Self Localization task adapted from [23], participants marked (through a single cross) the location of their ‘self’ on three female silhouettes (front, back and side view). Descriptive statistics are reported in Table 1.

The *second session* was the actual experimental session. It consisted of two tasks presented in counterbalanced order: the SELF and the FRIEND (control) task. Cardiac activity was registered using an Arduino compatible e-Health platform (Cooking Hacks). The software ExpVR (http://lnco.epfl.ch/expyvr) was used to present the visual stimuli and to record participants’ responses. First, the quality of the ECG recording was checked. Then, the models' full faces (original pictures with hair and ears) were shown on the monitor for 2 minutes. Participants, who had never seen the faces, were asked to memorize them during this time. Three practice trials were done (excluded from the analysis) to familiarize participants with the task.

At the end of the practice trials, participants performed the SELF and the FRIEND task in counterbalanced order. The SELF/FRIEND tasks were composed of three blocks, each associated to a different "Flashing Rate". Based on previous studies [14,15], the flashing rate was either ‘Synchronous’ or ‘Asynchronous’ (i.e. slower in half of the participants [90%] and faster in the other half [110%]) with the participants’ heartbeat. We added a third flashing rate as a further control, the ‘Heartbeat-Independent’, which was pre-defined for all participants and clearly too fast to be heartbeat-related (21 stimuli in 8 sec, equivalent to 157.5 bpm).

Each block consisted of 30 trials. Each trial was associated with a morphed stimulus and presented in randomized order. During each trial, the morphed face flashed on the screen for 8 seconds, after which participants were instructed to rate how much of the self- or friend- face, respectively, was present in each image, on a 0–100 (0 = “not me/my friend at all”; 100 = “totally me/my friend”) visual analogue scale (VAS). A random inter-trial interval (ITI) ranging from 100 to 500 ms was added after each participant's response.

A semi-structured interview was conducted at the end of the experiment in order to: 1) clarify whether participants had been aware of the manipulation and 2) to debrief them.

#### Data Handling

To provide a fine-grained analysis of the possible self-attribution bias induced by the synchronous visuo-cardio presentation, we fit the whole set of VAS scores attributed to the morphed images into a four-parameter sigmoid statistical model [which was based on the Boltzmann equation: y0 = A1 − A2/[1 + e(x−x0/dx)] + A2] for each subject and experimental condition (as in [12,27]). Appropriateness of the model was demonstrated for all conditions at the individual level (all Radjs ≥ 0.361; *p*s < .01), and the X0 values were extracted for each participant and condition. X0 value corresponds to the physical percentage of self/friend morphed values when participants subjectively rate the stimuli 50%-50%.

To check whether the different variants of the ‘asynchronous’ condition (i.e. fast or slow) influenced our findings, we performed two separate 3 ‘Flashing Rate’ (Synchronous, Asynchronous; Heartbeat-Independent) x 2 ‘Type of Asynchronicity’ (Asynchronous Fast; Asynchronous Slow) mixed-model ANOVAs on the dependent variable mentioned above. One was performed on the X0 values collected in the SELF task and the other one on those collected in the FRIEND task. After showing that the two types of asynchronicity did not affect the evaluation of the morphed faces, we combined the two in one single factor called ‘Asynchronous’. We then performed two separate (for SELF and FRIEND task) one-way analyses of variance (ANOVAs) with ‘Flashing Rate’ (Synchronous, Asynchronous; Heartbeat-Independent) as within-subjects factor. To test our main hypothesis, that is the effect of ‘Synchronicity’, we used two-tailed paired sample t-tests in which we compared X0 values collected in the Synchronous vs. Asynchronous conditions during both SELF and FRIEND tasks.

We performed three independent mixed models ANOVAs to test the additional hypothesis that participants with higher Interoceptive Accuracy and/or Interoceptive Sensibility, as well as those who localize themselves closer to the heart region, would have higher self-face attribution during Synchronous vs. Asynchronous flashing of the faces. The median split (0.678 ± 0.159; *M* ± *SD*) of the Counting task was used to divide participants into ‘High’ and ‘Low’ in Interoceptive Accuracy (IA). Thus, a 2 [IA group (‘High’ and ‘Low’ in IA)] x 2 [‘Synchronicity’ (Synchronous and Asynchronous)] ANOVA was run on the X0 values collected in the SELF task.

The median split (3.25 ± 0.887; *M* ± *SD*) of the Noticing sub-scale was used to divide participants in ‘High’ and ‘Low’ in Interoceptive Sensibility (IS). Thus, a 2 [IS group (‘High’ and ‘Low’ in IS)] x 2 [‘Synchronicity’ (Synchronous and Asynchronous)] ANOVA was run on the X0 values collected in the SELF task.

Finally, the results of the Self Localization task were used to divide participants in four groups: Hearters; Brainers, Belliers and ‘Other body-parts’. We only ran the analysis on the hearters and brainers because the other groups lacked an adequate number of participants (belliers = 4 and ‘non-body parts’ = 1).

Please see S1 Dataset for all relevant data concerning Study 1.

### Results

#### SELF and FRIEND tasks: Type of Asynchronicity

Results of the two separate 2 (“Type of Asynchronicity”) x 2 (“Flashing Rate”) ANOVAs performed on the X0 values collected in the SELF and FRIEND tasks show no significant main effects for the “Type of Asynchronicity” nor for the “Type of Asynchronicity” x “Flashing Rate” interactions (all *F*s < 0.183, all *p*s > .17). Following the results of this analysis we processed the two participant subgroups as belonging to the same group.

#### SELF and FRIEND tasks: Flashing Rate

**Fig 2:**
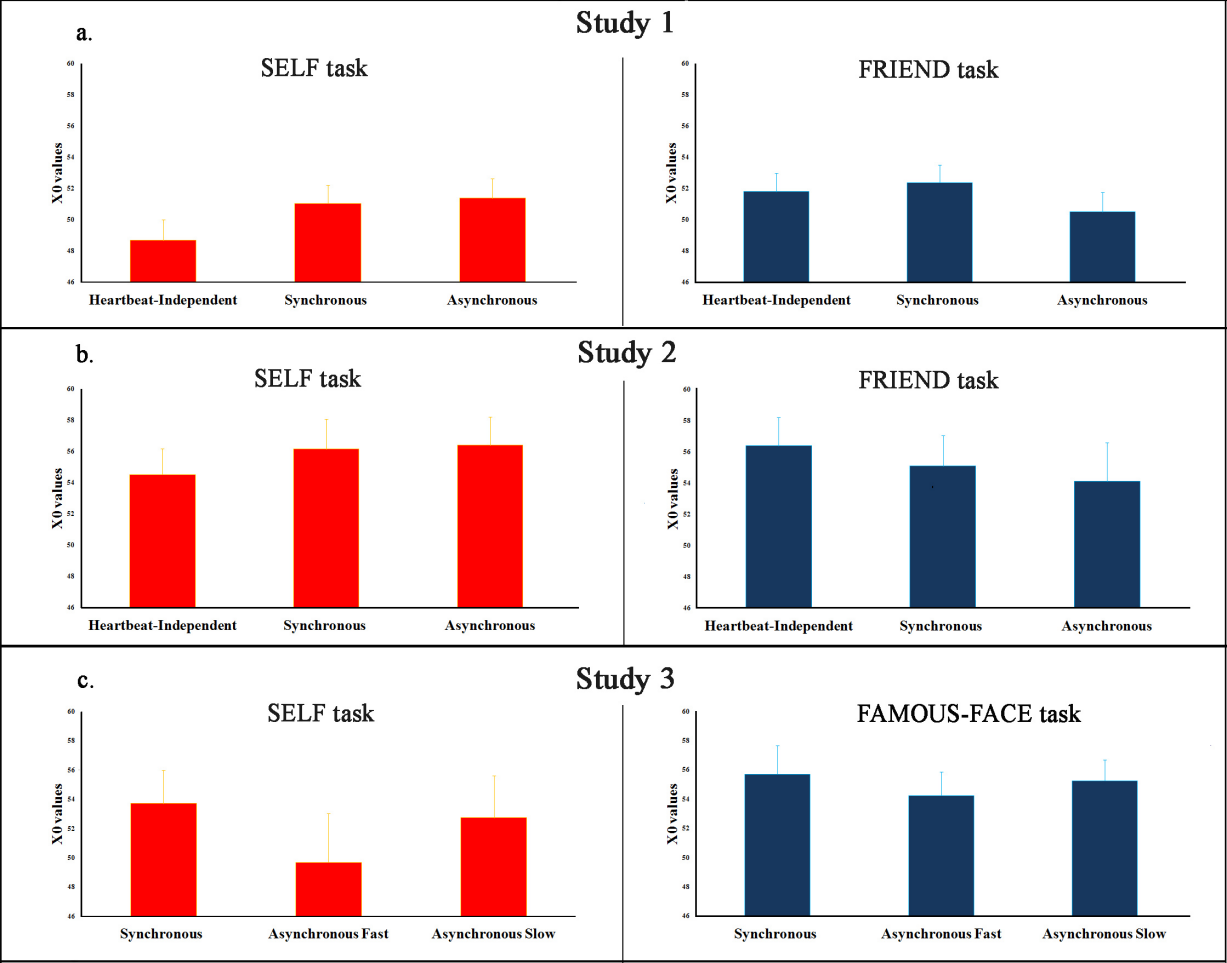
Summary of the main results. Panels a. and b. show the plots of X0 values collected in the SELF and in the FRIEND task in Study 1 and Study 2 respectively. Panel c. shows the plots of X0 values collected in the SELF and in the FAMOUS FACE task in Study 3.

Neither of the one-way ANOVAs (one within-subjects factor, i.e. “Flashing Rate”, on three levels: Synchronous, Asynchronous; Heartbeat-Independent) performed on the X0 values collected at the SELF and FRIEND task were significant (SELF: *F*(2, 62) = 2.875, *p* = .064; FRIEND: *F*(2, 62) = 1.703, *p* = .191), see Fig 2 panel a. for the plot of the data. This shows that the ‘Flashing Rate’ did not affect the participants’ judgments. As our main hypothesis was concerned with the difference between Synchronous and Asynchronous presentation, we looked further at the simple comparison between the two. The two-tailed t-tests were not significant for either of the two tasks (SELF: *t*(31) = -0.32; *p* = .75; FRIEND: *t*(31) = 1.79; *p* = .08). As classical null hypothesis testing is not the ideal statistical tool to make conclusions about non-significant results [30,31], we calculated Bayes Factors (BF) as well for our crucial comparison [SELF task: Synchronous vs. Asynchronous]. The Bayesian t-test (*BF* = .20) calculated in JASP [32] confirmed the null model results; namely, no differences were found between the Synchronous and Asynchronous conditions on the X0 values relative to the self-faces attribution.

#### SELF task: the splitting of participants according to Interoceptive Accuracy (IA)

Results of the mixed model ANOVA performed on the X0 values collected in the SELF task didn't show a main effect for the ‘IA group’ (‘High’ vs. ‘Low’ IA group) or a ‘Synchronicity’ (Synchronous vs. Asynchronous) x ‘IA group’ interaction (all *F*s < 1.813; all *p*s > .188).

#### SELF task: the splitting of participants according to Interoceptive Sensibility (IS)

Results of the mixed model ANOVA performed on the X0 values collected in the SELF task didn't show a main effect for the ‘IS group’ (‘High’ vs. ‘Low’ IS group) or a ‘Synchronicity’ (Synchronous vs. Asynchronous) x ‘IS group’ interaction (all *F*s < 1.13; all *p*s > .3).

#### SELF task: the splitting of participants according to the Self Localization task

Results of the mixed model ANOVA performed on the X0 values collected in the SELF task didn't show a main effect for the ‘Self Localization group’ (‘Hearters’ vs. ‘Brainers’) or a ‘Synchronicity’ (Synchronous vs. Asynchronous) x ‘Self Localization group’ interaction (all *F*s < 0.44; all *p*s *>*.51).

## Study 2

### Materials and Methods

Study 2 was designed to test whether the null results of Study 1 were due to our chosen asynchrony (90/110%) being too small for detection, and thus unable to influence participants’ performance. While this second study was methodologically identical to Study 1, asynchronicity was increased (to 75/125%) to make it more comparable to previous studies [14,15]. The study was approved by the local Ethics Committee of the Faculty of Arts and Social Sciences, University of Zurich.

#### Participants

An independent, naive sample of 18 female participants (22.61 ± 3.68 years; *M* ± *SD*) volunteered for Study 2, signed the informed consent and received a compensation for their participation. All but three participants were right-handed. None of them were aware of the visuo-cardiac synchronicity. In order to accept the null hypothesis in Study 2 as well, the stopping rule was defined by the BF being either higher than 0.3 (evidence for H1) or lower than 0.3 (evidence for H0), as this reveals substantial support for the Bayesian inference [30,31].

#### Data Handling

Data handling for the main analyses (i.e. the comparisons between the different ‘Flashing Rate’/‘Synchronicity’) performed on the X0 values measured in the SELF/FRIEND task was identical to Study 1.

Please see S1 Dataset for all relevant data concerning Study 2.

### Results

#### SELF and FRIEND tasks: Type of Asynchronicity

Congruently with Study 1, results of the 2 X 2 ANOVAs performed on the X0 values collected in the SELF and in the FRIEND task showed no significant main effects for the “Type of Asynchronicity” or “Type of Asynchronicity” x “Flashing Rate” interaction effects (all *F*s < 0.919, all *p*s > .351). These results authorized us to process the two participant subgroups as belonging to the same group, as we did in Study 1.

#### SELF and FRIEND tasks: Flashing Rate

Neither of the one-way ANOVAs performed on the X0 values collected in the SELF and in the FRIEND task were significant (SELF: *F*(2, 34) = 1.023, *p* = .37; FRIEND: *F*(2, 34) = 1.289; *p* = .289), showing that the ‘Flashing Rate’ did not affect the participants’ judgments, see Fig 2 panel b. for the plot of the data.

As our main hypothesis was concerned with the difference between Synchronous and Asynchronous presentation, we looked further at the simple comparison between the two. The two tailed t-tests were not significant for either of the two tasks (SELF: *t*(17) = -0.16; *p* = .87; FRIEND: *t*(17) = 0.63; *p* = .54). As classical null hypothesis testing is not the ideal statistical tool to make conclusions about non-significant results [29], we calculated Bayes Factors (BF) as well for our crucial comparison [SELF task: Synchronous vs. Asynchronous]. We replicated the null findings of Study 1, namely the BF of the mean comparison (SELF synchronous vs. SELF asynchronous) was 0.25 and thus in support of the null hypothesis (no difference between synchronous and asynchronous flashing in the SELF task).

#### SELF task: the splitting of the participants according to Interoceptive Accuracy (IA)

Results of the mixed model ANOVA performed on the X0 values collected in the SELF task didn't show a main effect for the ‘IA group’ (‘High’ vs. ‘Low’ IA group) or a ‘Synchronicity’ (Synchronous vs. Asynchronous) x ‘IA group’ interaction (all *F*s < 1.71; all *p*s > .29).

#### SELF task: the splitting of the participants according to Interoceptive Sensibility (IS)

Results of the mixed model ANOVA performed on the X0 values collected in the SELF task didn't show a main effect for the ‘IS group’ (‘High’ vs. ‘Low’ IS group) or a ‘Synchronicity’ (Synchronous vs. Asynchronous) x ‘IS group’ interaction (all *F*s < 1.213 all *p*s > .29).

#### SELF task: the splitting of the participants according to the Self Localization task

Results of the mixed model ANOVA performed on the X0 values collected in the SELF task didn't show a main effect for the ‘Self Localization group’ (‘Hearters’ vs. ‘Brainers’) or a ‘Synchronicity’ (Synchronous vs. Asynchronous) x ‘Self Localization group’ interaction (all *F*s < 0.11 all *p*s *>*.74).

## Study 3

### Materials and Methods

In Study 3 we extended the visuo-cardiac flashing of the faces to test whether the null results of Study 1 and Study 2 were due to an overly short (8 seconds per stimulus) presentation of the interoceptive signal (i.e. the heartbeat) combined with the visual stimuli (i.e. the morphed faces). A visuo-caridiac presentation of 2 minutes was used in the two studies [13,14] in which a significant increase in self-relatedness due to the visuo-cardiac synchronization was reported.

#### Participants

An independent, naive sample of 28 female participants (24.75 ± 3.86 years; *M* ± *SD*) volunteered for Study 3, signed the informed consent and received compensation for their participation. The stopping rule was defined by the BF being either higher than 0.3 (evidence for H1) or lower than 0.3 (evidence for H0) for the main comparison (SELF synchronous versus SELF asynchronous), as this reveals substantial support for the Bayesian inference [30,31].

Due to technical problems we excluded three participants. The final sample was thus composed of 25 participants (24.24 ± 2.47 years; *M* ± *SD*). All but one participant were right-handed. The study was approved by the local Ethics Committee of the Faculty of Arts and Social Sciences, University of Zurich.

#### Procedures

Study 3 also included two separate sessions. The *first session* resembled those of Study 1 and 2, though we did not measure Interoceptive Sensibility (see Table 1 again for descriptive statistics). Participants’ cardiac frequency (measured as beats per minute) was taken by using a mobile ECG device (ME 80, Beurer medical). The measured cardiac frequency was used to build the asynchronicity of the second session (see below). Furthermore, we showed the face of a famous woman (i.e. Melanie Winiger, a well known Swiss actress and model) to participants at the end of the first session, and, by using a standardized interview, asked them if they recognized the face. If they didn't, another famous face (i.e. Emma Watson, a well-known international actress) was presented and the same procedure was repeated (seven participants of the total sample). This famous face was then used as the stimulus in the FAMOUS FACE task performed in the second session. We used famous faces instead of friend's faces to simplify the experiment while still controlling for familiarity.

The *second session*, similar to Study 1 and 2, was the actual experimental session. It consisted of two main tasks presented in counterbalanced order: the SELF and the FAMOUS FACE task. Stimulus presentation and response registration were identical to Studies 1 and 2. The quality of the ECG recording was checked before starting. Each (SELF/FAMOUS) task consisted of 3 blocks that were presented in counterbalanced order across participants. Each block was associated with a different ‘visuo-cardiac flashing’, namely ‘Synchronous’ (flashing of the face was synchronized online with participant’s heart rate); ‘Asynchronous Fast’ (flashing of the face was 150% of the heart rate measured during the first session) and ‘Asynchronous Slow’ (flashing of the face was 50% of the heart rate measured during the first session). Each block consisted of 2 minutes of visuo-cardiac flashing: a selected morphed self-face (45% of self-face and 55% of the model-face) flashed in the SELF task and a selected morphed famous-face (45% of the famous-face and 55% of the model-face) flashed in the FAMOUS FACE task. Such morphing percentages were chosen based on a pilot study involving 12 participants (3 males) in which the three flashing rates (i.e. synchronous; asynchronous fast and asynchronous slow) were shown with a 100% self face, a morphed self-face (45% self– 55% unknown model-face) and a 100% unknown model-face. After the two-minutes of a/synchronous flashing, we measured how much self the shown picture contained on a 0–100 VAS. A trend obtained by running three separate Friedman ANOVAs (with ‘Flashing rate’ as the only within-subjects factor) shows that the only change between the a/synchronous visuo-cardiac flashing was the self-attribution ratings given to the morphed self face (*χ*2 = 4.667, *p* = .097), with a tendency to be higher in the synchronous condition. At the end of the visuo-cardiac flashing in the second session, similar to the pilot phase, participants were asked to evaluate how much of the self- or famous- face was present in the flashing stimulus on a 0–100 (0 = “not me/famous face at all”; 100 = “totally me/famous face”) VAS. A list of 12 morphed stimuli were then randomly presented. The randomly presented percentages of self face in the SELF task and famous face in the FAMOUS task were as follows: 1%; 31%; 35%; 39%; 43%; 47%; 53%; 57%; 61%; 65%; 69%, 99%. The complementary percentages contained in the morphed stimuli were composed of one of the two unknown models' faces (the same used in Study 1 and 2, counterbalanced across participants).

Participants were instructed to observe each morphed face stimulus for 2 seconds and to rate how much of the self- or famous- face was present in each image, on a 0–100 (0 = “not me/famous face at all”; 100 = “totally me/famous face”) visuo-analogue scale (VAS). A random inter-trial interval (ITI) ranging from 100 to 500 ms was added. At the end of the experiment a semi-structured interview was used to: 1) clarify whether our manipulation had been detected and 2) debrief participants.

#### Data Handling

Data handling of the main analysis performed on the X0 values extracted from the sigmoidal fitting of the VAS ratings given in the SELF/FAMOUS FACE task was similar to Study 1 and 2. We first performed two separate ANOVAs (one for the SELF and one for the FAMOUS FACE task) with ‘Synchronicity’ as the only within-subjects factor. Here, differently from Study 1 and 2, ‘Synchronicity’ had three levels, as all the participants saw the self- and famous- faces flashing “Synchronously”, “Asynchronously Fast” and “Asynchronously Slow” with respect to their heartbeat. Additionally, as in Study 1 and 2, we tested our main hypothesis by using Bayesian statistics which are insensitive to the optional stopping rules [31,33]. We could not run the above-mentioned analyses on one participant because the sigmoid fitting failed. Another participant was also excluded from these analyses because the X0 value in one condition was a clear outlier (i.e. more than 3 SD from the mean of the group for that condition). Moreover, similarly to Study 1 and 2 we performed two independent mixed models ANOVAs to test the additional hypothesis that participants with higher Interoceptive Accuracy as well as those who localize themselves closer to the heart region, would have higher self-face attribution during Synchronous vs. Asynchronous Fast and Slow flashing of the faces.

Beyond the analysis performed on the X0 values, we analyzed the VAS ratings given immediately after the a/synchronous flashing of the 45%-55% morphed stimulus presented in the SELF/FAMOUS task by using two separate ANOVAs (one for the SELF and one for the FAMOUS FACE task) with ‘Synchronicity’ (i.e. ‘Synchronous’, ‘Asynchronous Fast’ and ‘Asynchronous Slow’) as the only within-subjects factor.

Please see S1 Dataset for all relevant data concerning Study 3.

### Results

#### SELF and FAMOUS FACE tasks: X0 values

We replicated the null findings of Study 1 and 2 on the X0 values for both the SELF and FAMOUS FACE tasks (all *F*s < 1.251; all *p*s > .296), see Fig 2 panel c. for the plot of the data. Again, a *BF* of .3 supported the null model for the crucial comparison (no main effect for ‘Synchronicity’ in the SELF task).

#### SELF task: the splitting of the participants according to Interoceptive Accuracy (IA)

Results of the mixed model ANOVA performed on the X0 values collected in the SELF task didn't show a main effect for the ‘IA group’ (‘High’ vs. ‘Low’ IA group) or a ‘Synchronicity’ (Synchronous, Asynchronous Fast, Asynchronous Slow) x ‘IA group’ interaction (all *F*s < 1.44; all *p*s > .25).

#### SELF task: the splitting of the participants according to the Self Localization task

Results of the mixed model ANOVA performed on the X0 values collected in the SELF task didn't show a main effect for the ‘Self Localization group’ (‘Hearters’ vs. ‘Brainers’) or a ‘Synchronicity’ (Synchronous, Asynchronous Fast, Asynchronous Slow) x ‘Self Localization group’ interaction (all *F*s < 1.125 all *p*s *>*.34).

#### SELF and FAMOUS FACE tasks: VAS ratings

Neither of the ANOVAs performed on the VAS ratings given to the flashing 45%-55% morphed face of the SELF/FAMOUS FACE task were significant (all *F*s < 2.581; all *p*s > .086). This suggests that the morphed faces were similarly evaluated after the 2 minutes of visuo-cardiac a/synchronous flashing.

## Discussion

In three studies with independent samples of participants we investigated whether interoceptive signals (i.e. cardiac) coupled with exteroceptive ones (i.e. visual) might influence self-face attribution. We presented ambiguous self faces which flashed for 8 seconds (Study 1 and 2) or 2 minutes (Study 3). The flashing was either synchronous or asynchronous with participants' cardiac signaling (i.e. heartbeat). A friend's face was used as a control in Study 1 and 2, while in Study 3 a famous face was used.

After the flashing, participants were asked to evaluate how much of their own (or of the familiar face) was present in the ambiguous morphed faces. Contrary to our main hypothesis, self-face attribution was not higher after synchronous visuo-cardiac flashing than it was after asynchronous flashing. These results were supported by Bayesian statistics showing substantial evidence for H0.

We predicted that synchronous visuo-cardiac flashing would affect self-face attribution based on accumulating theoretical and empirical evidence suggesting that exteroceptive-interoceptive integration mechanisms underlie the bodily self and its plasticity (e.g. [34] for a recent review). Both the rubber hand illusion [17] and the enfacement illusion [16] have shown to vary in relation to the participants’ Interoceptive Accuracy, plausibly suggesting that individuals who are more aware of their internal signals, and rely more heavily on them, are less susceptible to exteroceptive illusions. In line with this, two pioneering studies [14,15] show that synchronous visuo-cardiac stimulations might directly influence body-ownership and self-location during the rubber hand [14] or full-body illusions [15], and that, contrary to the classical rubber hand illusion [17], the cardiac version of the illusion is stronger in people with high Interoceptive Accuracy [14].

Therefore, it could be hypothesized that self-attribution of the own face would be altered in a similar way by visuo-cardiac and visuo-tactile stimulation. Indeed, the experience of a synchronous visuo-tactile stimulation with another person, similar to the one used in the rubber hand illusion but experienced on the face, has previously been shown to blur self-other distinction by: i) altering a specific aspect of self-awareness, namely self-face recognition [12,13]; ii) increasing social similarity between self and others [35]; iii) canceling differences in remapping observed and perceived tactile stimuli onto one’s own somatosensory system [36] and iv) reducing the overwhelmingly distracting power of self-gaze [37].

Thus, although the hypothesis was strongly grounded in current literature, we did not find visuo-cardiac synchronicity to influence self-face attribution in the present three studies. Null results were found regardless of the duration of the visuo-cardiac stimulation as well as the degree of asynchronicity chosen as a control condition.

Our findings contradict the idea of visuo-cardiac synchronicity influencing all aspects of self-attribution that visuo-tactile synchronicity has been found to influence. It thus contributes to recent theoretical models on the role of interoceptive signals in the construction of bodily self, and specifically of self-face representation [18,19,38]. Given the general problem of publication bias and the fact that this is a new, vivacious, rapidly growing field of research, it is our strong opinion that these null findings will contribute to future research.

Currently we can only speculate on what underlies the null findings reported here. First, it could be that self-face attribution is a strongly *visual* process (thus relying more on exteroceptive signals) compared to the rather *multisensory* processes such as body-ownership and self-location. In line with the idea that self-face attribution relies on a visual representation, and that the visual representation is altered by multisensory illusions, Apps and collaborators [24] showed that between the crucial brain areas involved in the enfacement illusion, namely the right temporo-parietal junction (rTPJ) and intraparietal sulcus (IPS), the activity of unimodal regions such as the inferior occipital gyrus (IOG) was also modulated in accordance with the self-reported strength of the illusory experience. Indeed, it has been demonstrated that within IOG, in an area called Occipital Face Area (OFA), there are neurons that respond to a specific category of stimuli, such as faces. These neurons also process individual facial features more than the configural information contained in a face [39,40]. Therefore, according to the authors, synchronous visuo-tactile stimulations are able to trigger changes in unimodal representation of low-level visual features belonging to the seen faces because participants attribute the other's facial features to the mental representation of one’s own face [41]. Differently, neuroimaging studies investigating feelings of vicarious ownership and self-location (by using full-body illusion and rubber hand illusion paradigms) report the crucial activation of the premotor cortex, the intra-parietal sulcus (IP), the cerebellum and the TPJ [7,42,43], but not of visual areas. Only Ionta and collaborators [43] found a partial involvement of the extrastriate body area (EBA), but this activity did not map illusory feelings about bodily self-location, as it was not specific for the body being present, even in the control condition represented by an object. Thus it is possible that, differently from synchronous visuo-tactile stimulation, synchronous visuo-cardiac stimulation modulates the activity of multimodal areas involved in body-ownership and self-location, but not of unimodal areas necessary for the creation of self-face attribution bias such as the IOG.

It is worth noting that although self-face recognition is stable and, apart from severe neurological or psychiatric disorders [44], rarely disrupted, alterations in self-face attribution have been observed in the enfacement illusion [12,13]. However, it is also important to emphasize that the alterations induced by the enfacement illusion present lots of variability between participants, and that they are weaker compared to alterations reported on body ownership and self-location during rubber hand and full-body illusion paradigms. In line with this idea, a recent study by Estudillo and collaborators [45] shows that enfacement illusion did not report a significant reduction of racial bias, while Peck and collaborators [46] and Farmer and collaborators [47] published positive findings using full-body illusion and rubber hand illusion respectively.

Moreover, as also suggested by [14], interoceptive awareness is differently correlated to the various degrees of embodiment. For example, it positively correlates with the feeling of owning a body (as in the virtual version of the rubber hand paradigm), (see [14]) and negatively with the feeling of embodying an external body part (as in the classical rubber hand paradigm), (see [17]). Therefore, observing in third person perspective interoceptive signals coupled with more or less ambiguous self faces might be more incongruent than observing a virtual hand in first person perspective flashing synchronously with the heartbeat and thus induce no overestimation of self-face. Thus the latter may induce no overestimation of self-face.

There might also be differences in “Interoceptive Accuracy” (*M* = 0.64 vs. *M* = 0.77) between our participants and those tested by [14] who were sensitive to the effects of the visuo-cardiac stimulation. Our participants may not have been interoceptively accurate enough to be influenced by the visuo-cardiac synchrony.

These points could also help to understand other null findings relative to intero-exteroceptive full-body illusion, namely, the fact that Ronchi and collaborators [20] failed to replicate the influence of visuo-cardiac synchronicity on self-identification and self-location in a cardiac full-body illusion setup.

It is further worth noting that the effect of visuo-cardiac stimulation (in a comparable experimental setup) per se is weaker than the effect of visuo-tactile stimulation, as shown by [14]. This is not surprising given the fact that we often see our body parts touched, but rarely (at least consciously) see our body parts pulsating at the rhythm of our heart. This could be particularly true for the face that we only observe through a mirror.

## Conclusions

These three studies clearly suggest that, in contrast to hand and full-body attribution, visuo-cardiac synchronicity does not bias self-face attribution, at least not in the current experimental setup. The fact that visuo-cardiac stimulation might influence specific components of the bodily self makes this research area exciting, and we believe that empirical results, even if null, are of great help in the future.

Future experiments will be required to more fully investigate the bodily self's sensitivity to interoceptive signals and the related neural mechanisms. The extent to which exteroceptive and interoceptive body-related signals mutually influence one another in their successful contribution to the bodily self must also be determined.

## Supporting Information

S1 Dataset Dataset of Study 1, Study 2 and Study 3. S1 Dataset contains all relevant data concerning the three studies listed in the present manuscript.
(XLS)

## References

[1] Blanke O, Slater M, Serino A. Behavioral, Neural, and Computational Principles of Bodily Self-Consciousness. Neuron. Elsevier Inc.; 2015;88: 145–166. 10.1016/j.neuron.2015.09.029 26447578

[2] Botvinick M, Cohen J. Rubber hands “feel” touch that eyes see. Nature. 1998;391: 756 10.1038/35784 9486643

[3] Armel K, Ramachandran V. Projecting sensations to external objects: evidence from skin conductance response. Proc R Soc L B Biol Sci. 2003;270. 10.1098/rspb.2003.2364 PMC1691405 12965016

[4] Tieri G, Tidoni E, Pavone EF, Aglioti SM. Body visual discontinuity affects feeling of ownership and skin conductance responses. Sci Rep. 2015;5: 17139 10.1038/srep17139 26602036 PMC4658534

[5] Moseley GL, Olthof N, Venema A, Don S, Wijers M, Gallace A, et al Psychologically induced cooling of a specific body part caused by the illusory ownership of an artificial counterpart. Proc Natl Acad Sci U S A. 2008;105: 13169–73. 10.1073/pnas.0803768105 18725630 PMC2529116

[6] Barnsley N, McAuley JH, Mohan R, Dey a, Thomas P, Moseley GL. The rubber hand illusion increases histamine reactivity in the real arm. Curr Biol. Elsevier; 2011;21: R945–6. 10.1016/j.cub.2011.10.039 22153159

[7] Ehrsson HH, Spence C, Passingham RE. That’s my hand! Activity in premotor cortex reflects feeling of ownership of a limb. Science. 2004;305: 875–7. 10.1126/science.1097011 15232072

[8] Tsakiris M, Hesse MD, Boy C, Haggard P, Fink GR. Neural signatures of body ownership: a sensory network for bodily self-consciousness. Cereb Cortex. 2007;17: 2235–44. 10.1093/cercor/bhl131 17138596

[9] Lenggenhager B, Hilti L, Brugger P. Disturbed Body Integrity and the “Rubber Foot Illusion.” Neuropsychology. 2014; 10.1037/neu0000143 25265068

[10] Lenggenhager B, Tadi T, Metzinger T, Blanke O. Video Ergo Sum: Manipulating Bodily. Science (80-). 2007;317: 1096–1099. 10.1126/science.1143439 17717189

[11] Michel C, Velasco C, Salgado-Montejo A, Spence C. The Butcher’s Tongue Illusion. Perception. 2014;43: 818–824. 10.1068/p7733 25549512

[12] Sforza A, Bufalari I, Haggard P. My face in yours: Visuo-tactile facial stimulation influences sense of identity. Soc Neurosci. 2010; 10.1080/17470910903205503 19813138

[13] Tsakiris M. Looking for myself: current multisensory input alters self-face recognition. PLoS One. 2008;3: e4040 10.1371/journal.pone.0004040 19107208 PMC2603324

[14] Suzuki K, Garfinkel SN, Critchley HD, Seth AK. Multisensory integration across exteroceptive and interoceptive domains modulates self-experience in the rubber-hand illusion. Neuropsychologia. 2013;51: 2909–17. 10.1016/j.neuropsychologia.2013.08.014 23993906

[15] Aspell JE, Heydrich L, Marillier G, Lavanchy T, Herbelin B, Blanke O. Turning body and self inside out: visualized heartbeats alter bodily self-consciousness and tactile perception. Psychol Sci. 2013;24: 2445–53. 10.1177/0956797613498395 24104506

[16] Tajadura-Jiménez A, Tsakiris M. Balancing the “Inner” and the “Outer” Self: Interoceptive Sensitivity Modulates Self-Other Boundaries. J Exp Psychol Gen. 2013; 10.1037/a0033171 PMC3848898 23750913

[17] Tsakiris M, Tajadura-Jiménez A, Costantini M. Just a heartbeat away from one’s body: interoceptive sensitivity predicts malleability of body-representations. Proc Biol Sci. 2011;278: 2470–2476. 10.1098/rspb.2010.2547 21208964 PMC3125630

[18] Damasio A. Self comes to mind: Constructing the conscious brain. Pantheon. New York; 2010.

[19] Craig AD. How do you feel? Interoception: the sense of the physiological condition of the body. Nat Rev Neurosci. 2002;3: 655–66. 10.1038/nrn894 12154366

[20] Ronchi R, Bello-Ruiz J, Lukowska M, Herbelin B, Cabrilo I, Schaller K, et al Right insular damage decreases heartbeat awareness and alters cardio-visual effects on bodily self-consciousness. Neuropsychologia. Elsevier; 2015;70: 11–20. 10.1016/j.neuropsychologia.2015.02.010 25676677

[21] Garfinkel SN, Critchley HD. Threat and the Body: How the Heart Supports Fear Processing. Trends Cogn Sci. Elsevier Ltd; 2016;20: 34–46. 10.1016/j.tics.2015.10.005 26628111

[22] Garfinkel SN, Seth AK, Barrett AB, Suzuki K, Critchley HD. Knowing your own heart: Distinguishing interoceptive accuracy from interoceptive awareness. Biol Psychol. 2015;104: 65–74. 10.1016/j.biopsycho.2014.11.004 25451381

[23] Limanowski J, Hecht H. Where Do We Stand on Locating the Self? Psychology. 2011;02: 312–317. 10.4236/psych.2011.24049

[24] Apps MAJ, Tajadura-Jiménez A, Sereno M, Blanke O, Tsakiris M. Plasticity in Unimodal and Multimodal Brain Areas Reflects Multisensory Changes in Self-Face Identification. Cereb Cortex. 2013;1: [Epub ahead of print]. 10.1093/cercor/bht199 PMC4415063 23964067

[25] Faul F, Erdfeld E, Lang AG, Buchner A. A flexible statistical power analysis program for the social, behavioral, and biomedical sciences. Behav Res Methods. 2007;39: 175–191. 17695343 10.3758/bf03193146

[26] Cohen J. Statistical power analysis for the behavioral sciences 2nd ed. Hillsdale, editor. NJ: Erlbaum; 1988.

[27] Bufalari I, Lenggenhager B, Porciello G, Serra Holmes B, Aglioti SM. Enfacing others but only if they are nice to you. Front Behav Neurosci. 2014;8: 1–12. 24734011 10.3389/fnbeh.2014.00102 PMC3975105

[28] Schandry R. Heartbeat perception and emotional experience. Psychophysiology. 1981;18: 483–488. Available: 10.1111/j.1469-8986.1981.tb02486 7267933

[29] Mehling WE, Price C, Daubenmier JJ, Acree M, Bartmess E, Stewart A. The Multidimensional Assessment of Interoceptive Awareness (MAIA). PLoS One. 2012;7: e48230 10.1371/journal.pone.0048230 23133619 PMC3486814

[30] Dienes Z. Using Bayes to get the most out of non-significant results. Front Psychol. 2014;5 10.3389/fpsyg.2014.00781 PMC4114196 25120503

[31] Dienes Z. How Bayes factors change scientific pratice. J Math Psychol. Elsevier Inc.; 2015; 1–29. 10.1007/s13398-014-0173-7.2

[32] Love J, Selker R, Marsman M, Jamil T, Dropmann D, Verhagen A J, et al. JASP (Version 0.6.6). [Computer software]. 2015.

[33] Jeffreys H. The Theory of Probability. 3rd ed. Clarendon, editor. 1961.

[34] Brugger P, Lenggenhager B. The bodily self and its disorders. Curr Opin Neurol. 2015;27: 644–652. 10.1097/WCO.0000000000000151 25333602

[35] Paladino M-P, Mazzurega M, Pavani F, Schubert TW. Synchronous multisensory stimulation blurs self-other boundaries. Psychol Sci. 2010;21: 1202–7. 10.1177/0956797610379234 20679523

[36] Cardini F, Tajadura-jiménez A, Serino A, Tsakiris M. It feels like it ‘ s me: interpersonal multisensory stimulation enhances visual remapping of touch from other to self. J Exp Psychol Hum Percept Perform. 2013;44 10.1037/a0031049.It PMC3750640 23276110

[37] Porciello G, Holmes BS, Liuzza MT, Crostella F, Aglioti SM, Bufalari I. Interpersonal Multisensory Stimulation reduces the overwhelming distracting power of self-gaze: psychophysical evidence for “engazement.” Sci Rep. 2014;4: 6669 10.1038/srep06669 25327255 PMC5377579

[38] Seth AK. Interoceptive inference, emotion, and the embodied self. Trends Cogn Sci. 2013;17: 565–73. 10.1016/j.tics.2013.09.007 24126130

[39] Barton JJS. Structure and function in acquired prosopagnosia: lessons from a series of 10 patients with brain damage. J Neuropsychol. 2008;2: 197 10.1348/174866407X214172 19334311

[40] Kanwisher N, Barton J. The functional architecture of the face system: integrating evidence from fMRI and patient studies In: Haxby J, Johnson M, Rhodes G, Calder A, editors. Handbook of face perception. Oxford: Oxford University Press, p 111–130. 2011.

[41] Tajadura-Jiménez A, Grehl S, Tsakiris M. The other in me: interpersonal multisensory stimulation changes the mental representation of the self. PLoS One. 2012;7: e40682 10.1371/journal.pone.0040682 22866177 PMC3404924

[42] Petkova VI, Björnsdotter M, Gentile G, Jonsson T, Li T-Q, Ehrsson HH. From part- to whole-body ownership in the multisensory brain. Curr Biol. 2011;21: 1118–22. 10.1016/j.cub.2011.05.022 21683596

[43] Ionta S, Heydrich L, Lenggenhager B, Mouthon M, Fornari E, Chapuis D, et al Multisensory mechanisms in temporo-parietal cortex support self-location and first-person perspective. Neuron. Elsevier Inc.; 2011;70: 363–74. 10.1016/j.neuron.2011.03.009 21521620

[44] Feinberg T, Keenan J. The Lost Self: Pathologies of the Brain and Identity. Oxford: Press, 255 Oxford. University; 2005.

[45] Estudillo AJ, Bindemann M. Multisensory stimulation with other-race faces and the reduction of racial prejudice. Conscious Cogn. Elsevier Inc.; 2016;42: 325–339. 10.1016/j.concog.2016.04.006 27129077

[46] Peck TC, Seinfeld S, Aglioti SM, Slater M. Putting yourself in the skin of a black avatar reduces implicit racial bias. Conscious Cogn. Elsevier Inc.; 2013;22: 779–787. 10.1016/j.concog.2013.04.016 23727712

[47] Farmer H, Tajadura-Jiménez A, Tsakiris M. Beyond the colour of my skin: how skin colour affects the sense of body-ownership. Conscious Cogn. Elsevier Inc.; 2012;21: 1242–56. 10.1016/j.concog.2012.04.011 22658684 PMC3772504

